# Perception and kairomonal response of the coccinellid predator (*Harmonia axyridis*) to the fall armyworm (*Spodoptera frugiperda*) sex pheromone

**DOI:** 10.3389/fphys.2023.1167174

**Published:** 2023-04-10

**Authors:** Yidi Zhan, Jiaojiao Wang, Xiaona Kong, Yong Liu

**Affiliations:** College of Plant Protection, Shandong Agricultural University, Taian, Shandong, China

**Keywords:** chemical cue, predatory beetle, Spodoptera frugiperda, odorant-binding protein, population adaptability

## Abstract

Pheromone cues released from hosts or prey are of crucial importance to natural enemies for prey and habitat location. The use of herbivorous insect sex pheromones has long been considered as a potential pest control alternative that is non-toxic and harmless to beneficials. We hypothesized that *Harmonia axyridis* (Pallas) (Coleoptera: Coccinellidae), a major predatory coccinellid beetle of the devastating migratory pest *Spodoptera frugiperda* (Smith) (Lepidoptera: Noctuidae), could perceive and use the sex pheromone of *S. frugiperda* to locate its habitat. Here we tested the electrophysiological and behavioral responses of *H. axyridis* to the two components Z7-12:Ac and Z9-14:Ac of *S. frugiperda* sex pheromone by using electroantennography (EAG) and Y-tube bioassay. The 3D modeling of *H. axyridis* odorant-binding proteins (HaxyOBPs) and molecular docking were also performed. The results showed that both female and male *H. axyridis* exhibited significantly higher electrophysiological and behavioral responses to Z9-14:Ac at the concentrations of 0.001, 0.01, and 0.1 μg/μL, while no significant electrophysiological and behavioral responses of *H. axyridis* were observed to Z7-12:Ac. The blend of Z7-12:Ac and Z9-14:Ac at the ratio of 1:100 had a significant attraction to both male and female *H. axyridis* at the concentrations of 0.01 and 0.1 μg/μL based on electrophysiological and behavioral assays, but no significant behavioral responses were observed at the ratios of 1:9. According to the 3D modeling of HaxyOBPs and molecular docking, HaxyOBP12 has a good affinity with Z9-14:Ac. Z9-14:Ac is bound to the HaxyOBP12 by hydrogen bonding and hydrophobic interactions. However, there were no credible docking results between HaxyOBPs and Z7-12:Ac. Our findings revealed that *H. axyridis* can perceive Z9-14:Ac and could use it as a chemical cue to locate prey habitat. We speculated that Z7-12:Ac, which showed some antagonistic effect toward the response of *H. axyridis* to Z9-14:Ac, could improve the adaptability of *S. frugiperda* in the presence of predators. This study provides new insights into the application of pheromones to manipulate natural enemy behavior for pest control.

## 1 Introduction

Foraging behavior is a process in which insect natural enemies search for food resources or oviposition sites for survival, growth and reproduction (Kramer, 2001). Natural enemies of herbivores base their foraging decision on chemical cues from plant or herbivorous insects (Kaiser et al., 2017; Peñaflor, 2019). Pheromones released by hosts or prey may serve as kairomonal cues for parasitoids or predators, which can be used as kairomones for natural enemies to locate and control pests (Vaello et al., 2017). Common chemical signals released by the host or prey mainly include sex pheromones (Boo and Yang, 2000; Aukema and Raffa, 2005; Branco et al., 2006; Pekas et al., 2015; Shapira et al., 2018; Wang et al., 2018), alarm pheromones (Pickett et al., 2013; Wang et al., 2019; Liu et al., 2021; Qin et al., 2022), and aggregation pheromones (Kpongbe et al., 2019). To date, pheromone use is a promising way not only to monitor insect pests but to suppress their population growth by improving natural control function in agro-ecosystem.

Insects rely on sensitive olfactory systems to perceive chemical cues. Odorant-binding proteins (OBPs) are responsible for connecting the external environment and odorant receptors (ORs) (Brito et al., 2016). The interaction between OBPs and odor molecules is the first step in insect recognition of chemicals (Leal, 2012). The recognition mechanism of chemical signals of hosts or prey in natural enemies has been investigated within insect olfactory system. For example, aphid alarm pheromone E-β-Farnesene (EβF) was used as a foraging cue for many predators, such as hoverflies (Harmel et al., 2007; Wang et al., 2019), ground beetles (Kielty et al., 1996), green lacewings (Li et al., 2017; Li et al., 2018) and lady beetles (Liu et al., 2014).

Environmentally friendly pest management strategies including the use of natural enemies and pheromones are advocated. For example, slow release of aphid alarm pheromone EβF in wheat fields increases the abundance of mummified aphids by attracting parasitic wasps (Liu et al., 2021). However, the simultaneous use of these two methods may cause synergistic or antagonistic effects. The attraction of natural enemies to pheromones may enhance or interfere with biological control function when the natural enemies are able to generate kairomonal activity towards the pest pheromones (Shapira et al., 2018). Sex pheromone cues may arrest natural enemies to the source and enhance their foraging in the vicinity, thus contributing to the efficiency of pest control (Shapira et al., 2018). Alternatively, sex pheromone may attract natural enemies to the dispensers and reduce their densities in the field, thereby affecting the effectiveness of biological control (Pekas et al., 2015). Knowledge of the kairomonal effects of pheromones on enemies can help to improve lures that recruit and retain natural enemies and to improve the efficiency of biological control of crop pests (Ayelo et al., 2021; Ayelo et al., 2022).

The fall armyworm *Spodoptera frugiperda* (Smith) (*Lepidoptera: Noctuidae*) is a devastating agricultural pest, spreading rapidly in China and causing substantial economic losses (Wang et al., 2020; Wu et al., 2021). Management of *S. frugiperda* relies on the use of chemical insecticides, extracts and metabolites from plants, entomopathogenic bacterium, natural enemies, and sex pheromone traps (Paredes-Sánchez et al., 2021). *Harmonia axyridis* (Pallas) (Coleoptera: Coccinellidae) is a dominant predator that contributes to the suppression of many pests, including various hemipterans, and the larvae and pupae of Coleoptera, Hymenoptera, Diptera, and Lepidoptera (Cheng et al., 2020; Di et al., 2021). *H. axyridis* is a highly voracious predator of the eggs and young larvae of *S. frugiperda*, and can be used as biocontrol agent of this pest (Di et al., 2021). The use of monitoring based on pheromone traps has been shown to be effective to predict the infestation of *S. frugiperda* (Paredes-Sánchez et al., 2021). To date, Z9-14:Ac and Z7-12:Ac are identified as the two principal sex pheromone components of the Yunnan population of *S. frugiperda* (Jiang et al., 2022). However, the functions of these two components on natural enemies remain elusive. So we hypothesized that *H. axyridis* could recognize and detect *S. frugiperda* sex pheromone cues and recruit these cues for prey habitat location and prey location.

Here, we tested the electrophysiological and behavioral responses of *H. axyridis* to the two components Z7-12:Ac and Z9-14:Ac of *S. frugiperda* sex pheromone by using electroantennography (EAG) and Y-tube bioassay. Further tests were performed on the blend of Z7-12:Ac and Z9-14:Ac at the ratios of 1:9 and 1:100 to determine the response of *H. axyridis* to the binary mixture. Subsequently, the binding mechanism between the two pheromone components and *H. axyridis* odorant-binding protein (HaxyOBPs) has been clarified. The potential use of sex pheromone to manipulate the foraging behavior of *H. axyridis* for *S. frugiperda* control in the crops was discussed.

## 2 Materials and methods

### 2.1 Insects

Colonies of *H. axyridis* were collected from corn fields at the experimental station of Shandong Agricultural University (Tai’an, Shandong Province, China). Sex determination of *H. axyridis* was based on the labrum pigmentation (female: dark; male: light) and the distal margin of the 5th visible abdominal sternite (female: convex; male: concave) (McCornack et al., 2007). The lady beetles were reared with the wheat aphid *Sitobion miscanthi* (Takahashi) in the laboratory and maintained at 25°C ± 2°C and 65% ± 5% r. h under 12:12 L:D photoperiod. All *H. axyridis* used in this study were naive, and used only once. Before each trial, *H. axyridis* adults were starved for 48 h to enhance their sensitivity to odors.

### 2.2 Chemicals

(Z)-9-tetradecenal acetate (Z9-14:Ac) and (Z)-7-dodecenyl acetate (Z7-12:Ac) with minimum purity >90% were purchased from Shanghai Yuanye Biotechnology Co., Ltd. n-hexane (≥98%) was purchased from Kaitong Chemical Reagents Co., Ltd., Tianjin, China.

### 2.3 Comparative EAG responses to the sex pheromone of *S. frugiperda*

The dose-dependent EAG responses of male and female antennae to synthetic standard chemical compounds, Z7-12:Ac, Z9-14:Ac were investigated. According to AndoLab-PheromoneDatabase, the ratios of the pheromone components of *S. frugiperda* population that broke out in mainland China and east Asia were identified and determined as 1:9 or 1:100, so the blends of Z7-12:Ac and Z9-14:Ac at the ratios of 1:9 and 1:100 were also tested. Five concentrations of each component and the blends (each compound/blend was diluted with n-hexane into solutions of different gradient concentrations at 0.00001 μg/μL, 0.0001 μg/μL, 0.001 μg/μL, 0.01 μg/μL, and 0.1 μg/μL) were tested. Six different antennae were tested as replications for each concentration. Each antenna was stimulated 3 times. The antennae of *H. axyridis* were removed from the base, and the distal tips were also removed. Each dissected antenna was immediately fastened with electrode gel (Spectra 360 Electrode Gel) onto two metal electrodes. Ten microliter of each chemical solution was applied to a piece of filter paper strip (0.8 × 1 cm). The solvent was allowed to evaporate for 1 min, then the paper strip was placed inside a glass Pasteur pipette (5 cm in length, 0.5 cm in internal diameter) in the EAG system directed at the antennal preparation.

The stimuli were provided as 0.5 s puffs of air into a humidified air flow at 0.4 mL/s generated by a stimulus controller. A 15 s interval between successive stimulations was allowed for antennal recovery. EAG response to 10 μL n-hexane was tested as control. Signals were stored and analyzed by using EAG ver. 2.5 software (Synthech, Hilversum, Netherlands).

### 2.4 Y-tube olfactometer bioassay

The attractiveness of the sex pheromone of *S. frugiperda* to *H. axyridis* was assessed in dual choice assays using a Y-tube olfactometer (common arm, 20.0 cm; arms, 15 cm at 75°angle; internal diameter, 1.5 cm). Air was pumped through an active charcoal filter and re-humidified by passing it through a bottle with distill water before being directed into the two arms of the olfactometer. Chemicals and the blends were prepared in different concentrations (0.00001, 0.0001, 0.001, 0.01 and 0.1 μg/μL) and n-hexane was used as control. An aliquot (10 μL) of each test solution was applied to a filter paper strip (2 × 1 cm^2^), and the solvent was allowed to evaporate for 1 min before inserting the strip into an odor-source glass bottle connected to one arm of the olfactometer. The control glass bottle connected to the other arm of the olfactometer contained a filter paper strip treated with 10 μL of hexane. Adult *H. axyridis* was recorded as having made a choice that crossed half of the arm within 5 min. Twenty-five females or males that made a choice were tested for each treatment, individuals making no response to either arm for 5 min were recorded but discarded. The olfactometer was rotated by 90° after each test to avoid any directional bias. The olfactometer was thoroughly washed and rinsed with acetone after five replicates.

### 2.5 3D modeling and molecular docking

The OBPs sequences of *H. axyridis* were identified by Qu et al. (2021). Two strategies were employed to predict the 3D structure of OBPs. The online program SWISS MODEL (Waterhouse et al., 2018) was used for predicting the OBPs that have >30% homology with the templates in the Protein Data Bank (http://www.rcsb.org/pdb). While the OBPs that have less than 30% homology were generated using a deep residual neural network trRosetta (https://yanglab.nankai.edu.cn/trRosetta; Yang et al., 2020). The final 3D models were assessed by Procheck, Verify_3D and ERRAT (http://services.mbi.ucla.edu/SAVES/).

The binding mode between HaxyOBPs and *S. frugiperda* sex pheromone was performed using Surflex-Dock suit SYBYL X 2.1.1. The results of binding mode were evaluated according to the total score. The PyMOL and LigPlot^+^ (Laskowski and Swindells, 2011) were used to visualize conformations and interactions.

### 2.6 Data analysis

Data on EAG responses to each concentration of the sex pheromone components were analyzed using analysis of variance (ANOVA followed by LSD test). Preference numbers of Y-tube olfactory bioassay were evaluated by χ^2^ test. Individuals that did not make a choice were excluded from the statistical analysis.

## 3 Results

### 3.1 EAG response of *H. axyridis* to *S. frugiperda* pheromone

**FIGURE 1:**
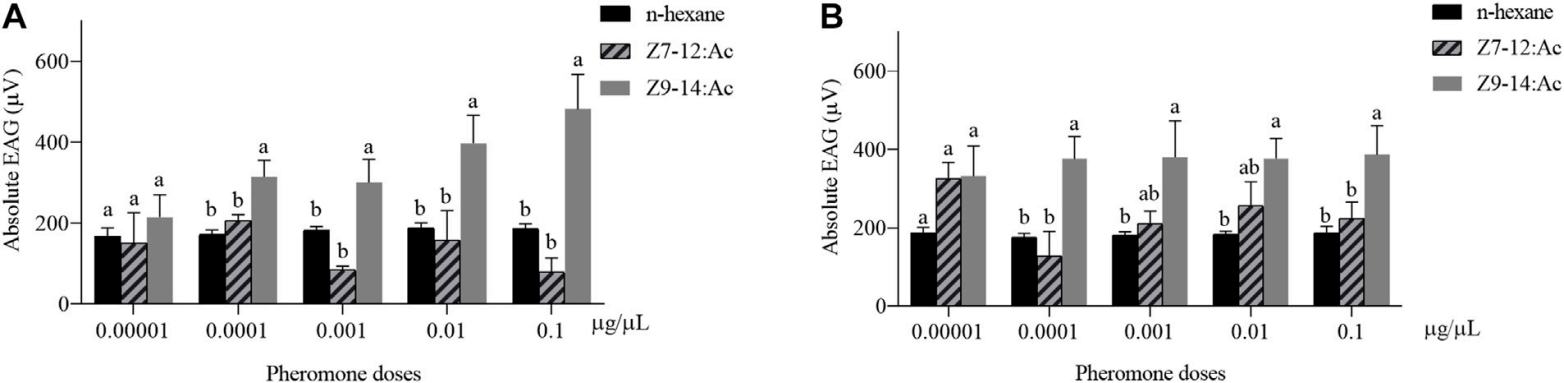
Electroantennogram (EAG) responses of male **(A)** and female **(B)** *Harmonia axyridis* to Z7-12:Ac and Z9-14:Ac with various doses (0.00001–0.1 μg/μL). Six different antennae were tested as replications for each concentration. Different letters indicate significant differences between different compounds (*p* < 0.05) at each concentration.

The dose-EAG responses of male and female antennae of *H. axyridis* to Z7-12:Ac and Z9-14:Ac were measured. Z9-14:Ac elicited higher EAG responses in both females and males than those of Z7-12:Ac (Figure 1). The concentration of 0.0001–0.01 μg/μL Z9-14:Ac caused significantly higher EAG responses in the males than those of Z7-12:Ac and n-hexane (0.0001 μg/μL: F = 7.072, df = 2, *p* = 0.007; 0.001 μg/μL: F = 9.884, df = 2, *p* = 0.002; 0.01 μg/μL: F = 4.971, df = 2, *p* = 0.022; 0.1 μg/μL: F = 14.782, df = 2, *p* < 0.001) (Figure 1A). Females showed significantly higher EAG responses to Z9-14:Ac at the concentrations of 0.0001 and 0.1 μg/μL than those of Z7-12:Ac and n-hexane (0.0001 μg/μL: F = 6.956, df = 2, *p* = 0.007; 0.1 μg/μL: F = 4.682, df = 2, *p* = 0.026) (Figure 1B).

**FIGURE 2:**
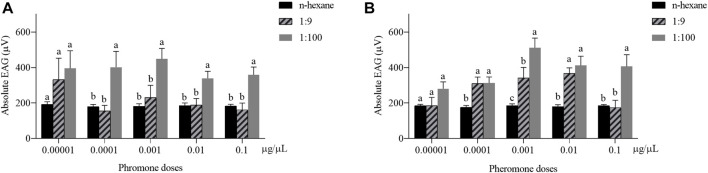
Electroantennogram (EAG) responses of male **(A)** and female **(B)** *Harmonia axyridis* to the blend of Z7-12:Ac and Z9-14:Ac at the ratios of 1:100 and 1:9. Six different antennae were tested as replications for each concentration. Different letters indicate significant differences at each concentration (*p* < 0.05).

The EAG response of *H. axyridis* to the blend of Z7-12:Ac and Z9-14:Ac at the ratio of 1:100 was higher than that to 1:9 (Figure 2). The responses of male to the ratio of 1:100 were significantly higher than those to 1:9 at the concentration of 0.0001–0.1 μg/μL (0.0001 μg/μL: F = 5.793, df = 2, *p* = 0.012; 0.001 μg/μL: F = 6.975, df = 2, *p* = 0.007; 0.01 μg/μL: F = 7.426, df = 2, *p* = 0.006; 0.1 μg/μL: F = 9.564, df = 2, *p* = 0.002) (Figure 2A). The female responses to 1:100 were significantly higher than those to 1:9 at the concentrations of 0.001 and 0.1 μg/μL (0.001 μg/μL: F = 12.157, df = 2, *p* = 0.022; 0.1 μg/μL: F = 8.472, df = 2, *p* = 0.002) (Figure 2B).

### 3.2 Olfactory response of *H. axyridis* to *S. frugiperda* pheromone

**FIGURE 3:**
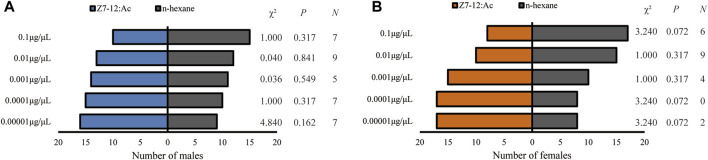
Olfactory responses of male **(A)** and female **(B)** *Harmonia axyridis* to Z7-12:Ac in a Y-tube olfactometer. Twenty-five females or males that made a choice were tested for each treatment. N: the number of non-responses.

**FIGURE 4:**
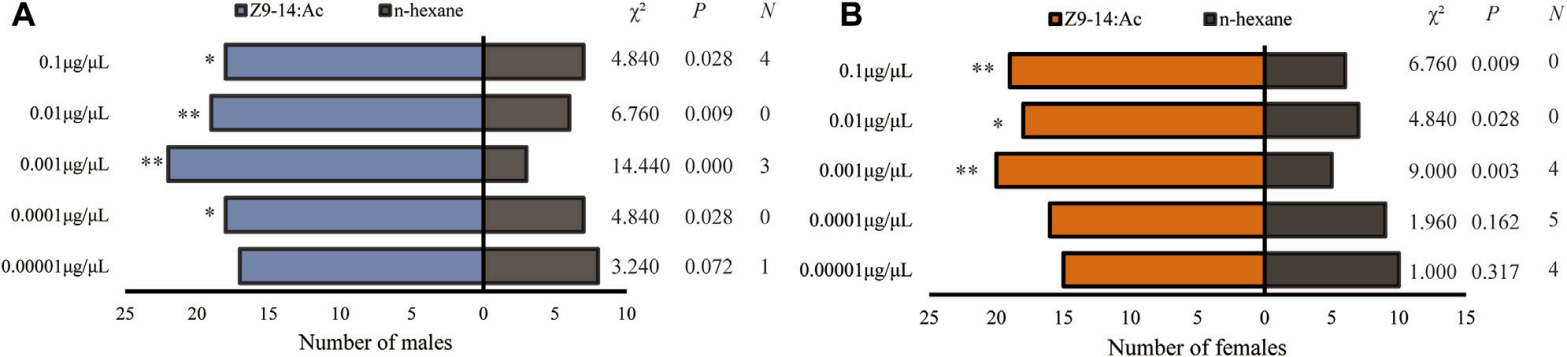
Olfactory responses of male **(A)** and female **(B)** *Harmonia axyridis* to Z9-14:Ac in a Y-tube olfactometer. Twenty-five females or males that made a choice were tested for each treatment. N: the number of non-responses. The asterisks indicate significance differences at *p* < 0.05 (*) and *p* < 0.01 (**).

Behavioral response tests showed that Z7-12:Ac elicited no significant responses of the male and female *H. axyridis* (Figure 3), while *H. axyridis* exhibited strongly attractive response to Z9-14:Ac (Figure 4)*.* At the concentrations ranging from 0.0001 to 0.1 μg/μL, Z9-14:Ac attracted significantly more males than that of n-hexane (Figure 4A), while females showed a significant tendency to Z9-14:Ac at the concentrations ranging from 0.001 to 0.1 μg/μL (Figure 4B).

**FIGURE 5:**
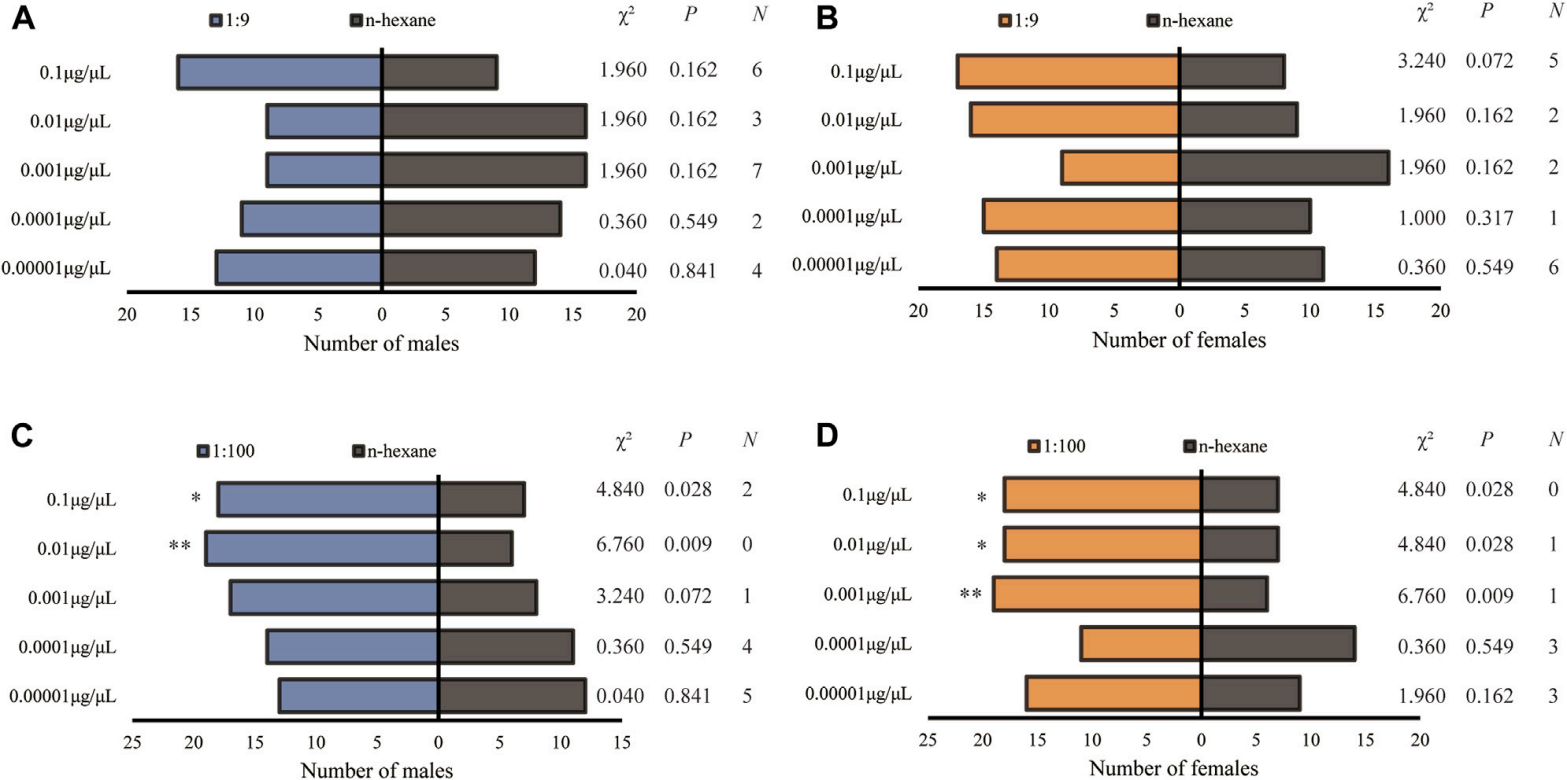
Olfactory responses of male and female *Harmonia axyridis* to the blend of Z7-12: Ac and Z9-14: Ac at the ratios of 1:9 (**(A)**: male; **(B)** female) and 1:100 (**(C)**: male; **(D)** female) in a Y-tube olfactometer. Twenty-five females or males that made a choice were tested for each treatment. N: the number of non-responses. The asterisks indicate significance differences at *p* < 0.05 (*) and *p* < 0.01 (**).

*H. axyridis* showed completely different olfactory responses to the blends of Z7-12:Ac and Z9-14:Ac at the ratios of 1:9 and 1:100. Neither male nor female showed a preference for a ratio of 1:9 (Figures 5A, B). Males displayed significant preferences for 0.01 and 0.1 μg/μL at the ratio of 1:100 (Figure 5C), and females did for 0.001, 0.01 and 0.1 μg/μL (Figure 5D).

### 3.3 3D modeling and molecular docking

**FIGURE 6:**
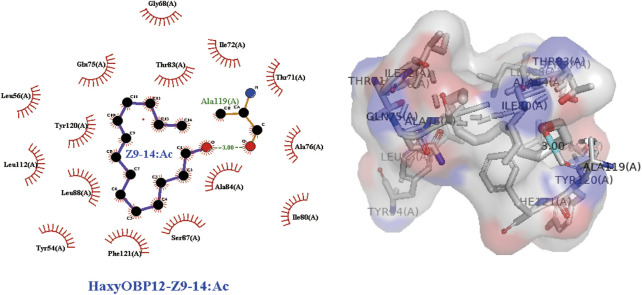
Interactions between HaxyOBP12 protein and Z9-14:Ac.

In order to take insight into the mechanism of the responses of *H. axyridis* to the two components of *S. frugiperda* sex pheromone, 3D modeling of *H. axyridis* odorant-binding protein (HaxyOBPs) and molecular docking were performed. Sequence alignments showed that HaxyOBP12, HaxyOBP13 and HaxyOBP14 shared more than 30% similarity with template proteins (Supplementary Table S1). In addition to these three OBPs, the other 16 OBPs used trRosetta for 3D structure prediction. The predicted results all matched very high (Supplementary Table S2). In order to provide evidence on whether *H. axyridis* could sense and detect *S. frugiperda* sex pheromone Z7-12: Ac and Z9-14: Ac, SYBYL was used to analyze the molecular interactions between identified HaxyOBPs and Z7-12:Ac or Z9-14:Ac respectively. The results revealed that HaxyOBP12 has a good affinity with Z9-14:Ac (Total score = 8.261, C-score = 4). However, there were no credible docking results between HaxyOBPs and Z7-12:Ac (Table 1). Z9-14:Ac bound to HaxyOBP12 by hydrogen bonding interactions and hydrophobic interactions (Figure 6). The Z9-14:Ac was stabilized by a hydrogen bond with Ala119 of HaxyOBP12 (3.00 Å). Additionally, Z9-14:Ac was also buried in tight hydrophobic cavities formed by residues including Thr83, Tyr120, Leu88, Phe121, Ser87, Ala84, Ile72, Gln75, Thr71, Gly68, Leu56, Leu112, Tyr54, Ile80, and Gly68.

**TABLE 1:** SYBYL docking results.

OBPs	Pheromone	Total score	Crash	Polar	D_score	PMF_score	G_score	Chemscore	C-score
1	Z7	7.4984	−1.3607	0.9609	−113.8115	−6.4901	−213.263	−19.3919	1
	Z9	7.3268	−0.8049	0.8704	−105.3687	26.9388	−222.5839	−18.2059	2
2	Z7	7.5051	−1.3151	0	−108.9621	2.8327	−194.4206	−22.3429	2
	Z9	7.2375	−1.213	1.1738	−102.3625	1.3023	−182.1999	−24.8529	0
3	Z7	5.7676	−2.0033	1.1022	−108.926	7.6937	−186.9444	−18.2901	1
	Z9	6.2581	−2.0055	2.2794	−96.7706	−13.1954	−202.9398	−20.9771	2
4	Z7	4.3342	−0.7624	0	−409.2545	13.2906	−133.6434	−24.8884	3
	Z9	5.0932	−0.5818	1.1844	−425.9345	10.2439	−141.6286	−20.2537	2
5	Z7	7.5756	−0.6529	1.0606	−106.1743	−26.0215	−180.9041	−18.0114	1
	Z9	7.2651	−1.7046	0.6805	−119.396	−1.9989	−227.8684	−23.2066	2
6	Z7	3.5491	−0.7891	1.1178	−77.9468	45.792	−120.6242	−7.3713	2
	Z9	4.4798	−1.1393	2.0942	−73.4433	43.5507	−115.8393	−11.2191	2
7	Z7	7.4842	−0.6505	2.0904	−103.6945	−69.1741	−194.3866	−24.1644	1
	Z9	6.9391	−0.8437	1.3879	−101.1283	−43.8382	−174.9243	−27.272	3
8	Z7	3.339	−1.1489	1.4246	−65.5465	−3.9523	−133.2273	−14.7797	1
	Z9	6.5332	−0.5552	2.5324	−72.0579	−10.2369	−133.3388	−15.2353	1
9	Z7	8.3355	−1.6168	1.3211	−111.1679	−30.8611	−228.47	−20.4871	3
	Z9	8.7695	−1.4592	1.4936	−101.8224	−8.3198	−184.291	−25.9148	1
10	Z7	5.6784	−0.74	1.1019	−90.4941	−33.8735	−162.3957	−19.1104	1
	Z9	5.1383	−1.0542	0	−87.6996	−45.977	−168.931	−23.5797	4
11	Z7	9.2743	−1.0351	0	−121.3123	−54.2514	−227.9555	−27.5486	2
	Z9	8.4632	−2.2112	0	−127.8641	−41.3831	−243.0555	−30.4844	0
12	Z7	7.8721	−3.5849	0	−141.6934	−0.3747	−265.4919	−24.683	3
	Z9	8.261	−2.8121	0.8586	−132.7201	−12.5932	−283.4571	−30.5738	4
13	Z7	7.3845-	−1.8577	2.5499	−94.8605	−7.4795	−184.6	−20.9695	4
	Z9	7.1707	−1.4493	3.4835	−97.4874	−45.7917	−211.037	−29.6366	2
14	Z7	6.9463	−1.2832	0	−96.0042	−22.8851	−203.0978	−17.8821	1
	Z9	5.648	−1.0494	1.0287	−83.3187	−45.7147	−193.2538	−22.8586	2
15	Z7	3.2736	−0.6834	0	−284.6613	−3.0613	−139.3636	−18.6232	2
	Z9	4.8112	−0.8832	1.4739	−304.3181	27.2172	−155.7341	−24.0912	2
16	Z7	7.3746	−1.5197	1.0328	−113.9439	−41.5512	−212.1562	−24.3665	2
	Z9	6.871	−1.563	1.2432	−116.9874	−32.7259	−218.7278	−26.9907	3
17	Z7	6.0605	−1.256	1.171	−97.6921	9.5303	−178.554	−17.05	4
	Z9	5.8333	−0.9551	0.9593	−92.552	7.787	−170.0882	−17.3433	0
18	Z7	5.7515	−2.4271	0	−102.0482	25.0117	−215.5874	−15.5994	0
	Z9	6.2869	−1.1689	1.7872	−91.0506	−5.9071	−154.5452	−21.8616	1
19	Z7	8.0585	−2.0295	0.7858	−118.7177	−24.6058	−232.5992	−23.1932	3
	Z9	7.0481	−1.8582	0	−110.9702	−31.4045	−203.4581	−25.354	3

## 4 Discussion

Chemical cues play a key role in mediating the interactions between natural enemies and their hosts or prey. The host or prey can release kairomones that differs from the plant background odors, thus providing the most reliable source of chemical information for natural enemies to detect host and prey in the natural environment (Rodriguez-Saona and Stelinski, 2009; Ayelo et al., 2022). In this study, we examined and determined the kairomonal effect of *S. frugiperda* sex pheromone on the coccinellid predator *H. axyridis*, and the binding properties of *H. axyridis* OBPs to the components of the sex pheromone. Our findings could provide new strategies for the combined application of sex pheromone and natural enemies for *S. frugiperda* control.

The pheromone composition of *S. frugiperda* was inconsistent among different geographic populations (Jiang et al., 2022). To sum up, Z9-14:Ac, Z7-12:Ac, Z9-12:Ac, Z11-16:Ac and E7-12:Ac were employed by different geographic populations (Sekul and Sparks, 1967; Sekul and Sparks, 1976; Tumlinson et al., 1986; Groot et al., 2008; Jiang et al., 2022). The sex pheromone components of the Yunnan population C-strain of *S. frugiperda* are confirmed as Z7-12:Ac and Z9-14:Ac (Jiang et al., 2022). Based on the pre-test of the EAG responses of *S. frugiperda* to the five identified sex pheromone components, we found that Z9-14:Ac and Z7-12:Ac elicited strong electrophysiological responses (Supplementary Figure S1). Therefore, these two components were as the putative candidates for the experiments.

Electrophysiological and olfactory response assays of *H. axyridis* demonstrated that both female and male *H. axyridis* were strongly attracted to Z9-14:Ac. Molecular docking showed that OBP12 and Z9-14:Ac could be closely bonded through hydrogen bonding and hydrophobic interaction, while there was no good docking effect between OBP and Z7-12:Ac. The transcript of *HaxyOBP12* is mainly restricted to adult antennae, implying a potential role in olfactory chemoreception (Qu et al., 2021). These results revealed that *H. axyridis* can sense the stimulation of Z9-14:Ac and transfer the stimulus to its central nervous system. It was suggested that the sex pheromone component Z9-14:Ac of *S. frugiperda* could be recognized by *H. axyridis* and was acted as an important kairomonal signal when *H. axyridis* prey on the eggs and larvae of *S. frugiperda*. Moreover, *HaxyOBP12* is abundant in the larval stage (Qu et al., 2022), indicating that it is also involved in the recognition of Z9-14:Ac by *H. axyridis* in the larval stage to locate prey.

Although *H. axyridis* showed a higher kairomonal response to Z9-14:Ac, further research is needed to determine whether it could be efficient to be used as a pheromone bait to attract and assemble local population of *H. axyridis* in the fields. Several enemies, such as *H. axyridis* (Verheggen et al., 2007), *Coccinella septempunctata* (Ninkovic et al., 2001), the hoverfly *Episyrphus balteatus* (Verheggen et al., 2008), and aphid parasitoids (Liu et al., 2021), are able to perceive the aphid alarm pheromone EβF and show attractant behavior. But in the field, EβF had no effect on the number of predator (Araneae, Opilionidae, Carabidae, Coccinellidae, Staphilinidae, Forficulidae, Syrphidae, Formicidae, Polistinae, Parasitiod, and Chrysopidae) visits to aphid colony or on predator patch residence times (Joachim & Weisser, 2015). Thus, while sex pheromones may have potential for application in agricultural pest management strategies, their ecological role as kairomone in natural settings is worth pondering. The dose-dependent response tests showed that *H. axyridis* seemed to respond to higher concentrations of Z9-14:Ac, suggesting that the potential effect of concentration on natural enemy populations must be taken into consideration when determining the most effective dose to use (Branco et al., 2006).

In addition, *H. axyridis* had a significantly higher response to the mixture of Z7-12:Ac and Z9-14:Ac at the ratio of 1:100 than to 1:9. It suggested that Z7-12:Ac might increase the avoidance effect of Z9-14:Ac on *H. axyridis*. We speculated that insect pest populations have probably evolved in producing a variety of components of sex pheromones with various ratios, thus not only forming reproductive isolation, but also avoiding natural enemy predation or parasite. Therefore, the ratio effect of the two components of *S. frugiperda* sex pheromone on natural enemies should be considered when it is applied for monitoring and control of *S. frugiperda*.

## Data Availability

The original contributions presented in the study are included in the article/Supplementary Material, further inquiries can be directed to the corresponding authors.
