# Supplementary material for: Perception and kairomonal response of the coccinellid predator (Harmonia axyridis) to the fall armyworm (Spodoptera frugiperda) sex pheromone
Source: Front Physiol. 2023 Apr 10;14:1167174. doi: 10.3389/fphys.2023.1167174 (PMC10123280; doi:10.3389/fphys.2023.1167174)
Supplement: Supplementary file 1 [file DataSheet1.docx]

Supplementary Material

**Perception and kairomonal response of the coccinellid predator (*Harmonia axyridis*) to the fall armyworm (*Spodoptera frugiperda*) sex pheromone**

**Yidi Zhan^1†^, Jiaojiao Wang^1†^, Xiaona Kong^1^, Yong Liu^1^***

*** Correspondence:** Yong Liu: [liuyong@sdau.edu.cn](mailto:liuyong@sdau.edu.cn)

**
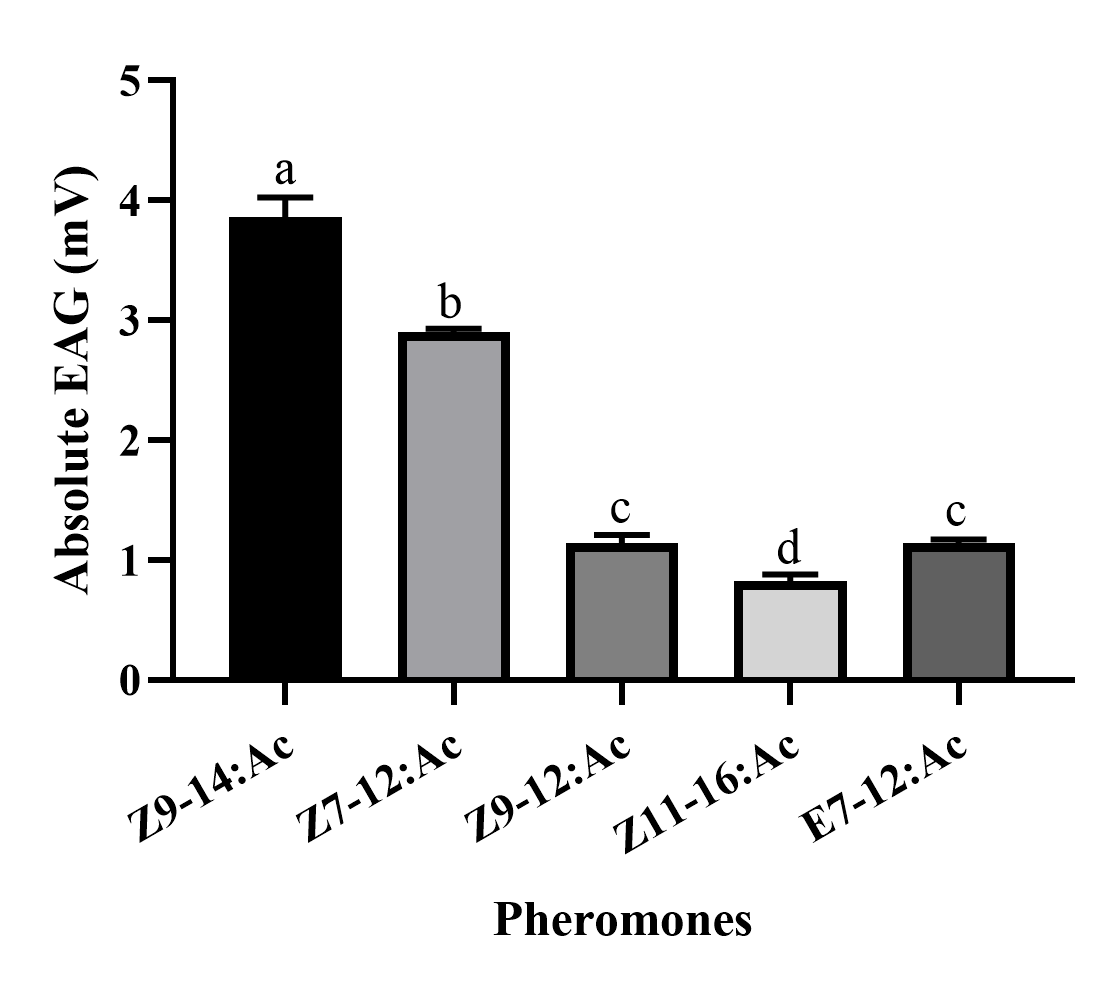
**

**Supplementary Figure 1** Electroantennogram (EAG) responses of male *Spodoptera frugiperda* to pheromone components (1 µg/µL, n=6).

**Supplementary Table 1** Homologous templates of odorant-binding proteins (OBPs) in *Harmonia axyridis* predicted by SWISS MODEL

| OBPs | Template | SMTL ID | Seq identity | Ramachandran favoured |
| --- | --- | --- | --- | --- |
| 12 | Crystal Structure of Odorant Binding Protein 4 in the Natural Predator *Chrysopa pallens* | 6jpm.1.A | 42.86 | 94.21 |
| 13 | Crystal Structure of Odorant Binding Protein 4 in the Natural Predator *C. pallens* | 6jpm.1.A | 30.7 | 96.52 |
| 14 | Structure of OBP3 from the vetch aphid *Megoura viciae* | 4z39.1.B | 30.48 | 94.50 |

**Supplementary Table 2** Estimated TM-scores of 3D model of odorant-binding proteins (OBPs) in *Harmonia axyridis* predicted by trRosetta

| OBPs | 1 | 2 | 3 | 4 | 5 | 6 | 7 | 8 |
| --- | --- | --- | --- | --- | --- | --- | --- | --- |
| Estimated TM-score | 0.708 | 0.874 | 0.729 | 0.613 | 0.895 | 0.785 | 0.813 | 0.830 |
| OBPs | 9 | 10 | 11 | 15 | 16 | 17 | 18 | 19 |
| Estimated TM-score | 0.688 | 0.721 | 0.774 | 0.888 | 0.835 | 0.679 | 0.750 | 0.766 |
